# Development and validation of a short and easy-to-use instrument for measuring health literacy: the Health Literacy Instrument for Adults (HELIA)

**DOI:** 10.1186/s12889-020-08787-2

**Published:** 2020-05-12

**Authors:** Mahmoud Tavousi, Aliasghar Haeri-Mehrizi, Fatemeh Rakhshani, Shahram Rafiefar, Atoosa Soleymanian, Fatemeh Sarbandi, Mona Ardestani, Shahla Ghanbari, Ali Montazeri

**Affiliations:** 1 Health Metrics Research Center, Iranian Institute for Health Sciences Research, ACECR, Tehran, Iran; 2 Department of Public Health, School of Public Health and Safety, Shahid Beheshti University of Medical Sciences, Tehran, Iran; 3 Health Education and Promotion Office, Deputy of Health, Ministry of Health and Medical Education, Tehran, Iran

**Keywords:** Health literacy, Psychometric properties, Adults, Questionnaire, HELIA

## Abstract

**Background:**

Health literacy is the ability to access to, understand, evaluate and use of essential health information to make basic health decisions. This study aimed to develop and psychometrically evaluate an instrument for measuring health literacy among adults (the Health Literacy Instrument for Adults - HELIA).

**Methods:**

In addition to a literature review, a panel of specialists from different disciplines was formed to generate an item pool. Then, a framework was defined to develop the initial questionnaire based on a definition of health literacy and the most important global public health issues. The initial questionnaire contained 66 items. Next, 15 experts in public health were approached to assess content validity. Consequently, 19 items were removed and a provisional version of the questionnaire with 47 items was provided. Finally, a random sample of adults completed the questionnaire and psychometric properties of the instrument were assessed.

**Results:**

Overall, 323 adults aged 18 to 65 years old completed the questionnaire. When the exploratory factor analysis was performed, 33 items were loaded, which indicated a 5-factor solution for the questionnaire that jointly explained 52.9% of the variance observed. The factors were as follows: access to information (6 items), reading (4 items), understanding (7 items), appraisal (4 items), and decision making/behavioral intention (12 items). Confirmatory factory analysis also indicated a good fit to the data for the five-latent structure (χ2/df = 1.60, SRMR = 0.049; RMSEA = 0.043; CFI = 0.98; NFI = 0.95; NNFI = 0.98 and GFI = 0.87). Additional analysis for internal consistency showed satisfactory results with Cronbach’s alpha coefficients ranging from 0.72 to 0.89. Intraclass correlation coefficient (test-retest analysis) also showed acceptable stability for the questionnaire (ICC = 0.84). The mean score for health literacy as measured by the HELIA was 76.3 (SD = 15.1) out of 100 for the study sample.

**Conclusion:**

The findings suggest that the Health Literacy Instrument for Adults (HELIA) is a valid and reliable instrument for measuring health literacy. It is a short and easy-to-use instrument that could be applied in different settings.

## Background

There is general agreement that health literacy goes beyond the ability to read, write and understand the meanings of words and numbers in health care settings [1]. Health literacy comprises various competencies and depends on individual and community factors. These factors include different issues ranging from cultural issues to health care, public health and other relevant systems and settings in which people obtain and use health information [2].

Health literacy is a complicated concept [3] that is a global issue [4] and its existence is a way to achieve good health. One common definition of health literacy is offered by the World Health Organization (WHO) and reads as follows: ‘the cognitive and social skills which determine the motivation and ability of individuals to gain access to, understand and use information in ways which promote and maintain good health’ [5]. It is believed that people with inadequate health literacy might suffer from poor health, have little information about disease prevention, participate less in clinical preventive services against chronic illnesses, and have trouble comprehending health instructions or interpreting them correctly [6, 7]. Furthermore, limited health literacy is associated with poor adherence to medical treatment and inappropriate communication with health professionals, more hospitalization, increased medical and health expenditure, higher mortality and morbidity and poorer self-care [8]. Therefore, the measurement of health literacy is an essential component of any effort to prevent consequences of limited health literacy and health care discrepancies [9].

The number of instruments developed to address and evaluate health literacy is growing rapidly. The most widely and frequently used instruments are the Rapid Estimate of Adult Literacy in Medicine (REALM) [10], the Test of Functional Health Literacy in Adults (TOFHLA) [11], and the Newest Vital Sign (NVS) [12]. These instruments have been criticized for several reasons, including for assessing only a few domains of health literacy, not being suitable for use in intervention studies or not having been developed with a health promotion perspective [13]. Furthermore, most of these scales were developed and used in clinical settings [14]. Haun et al. in a comprehensive review of the literature from 1999 through 2013 identified 51 instruments and reported that most instruments represent a narrow set of conceptual dimensions with limited modes of administration and missing information on key psychometric properties. They recommended that as researchers develop new measures, a full range of conceptual dimensions of health literacy and better validation studies should be included to establish sound evidence for measuring health literacy [15]. As such, instruments that were developed recently are improved greatly [16, 17]. More recently The WHO Regional Office for Europe provided a comprehensive review of older and the recent instruments (31 instruments) and concluded that at the policy level, frameworks and indicators that cover various domains are needed to enable consistent and comparable population monitoring and evaluations [18].

However, it should be noted that there are three types of health literacy instruments: general health literacy instruments, condition (disease or content) specific measures, and instruments that are developed for specific populations [15]. The WHO Regional Office for Europe also followed a very similar categorization for health literacy instruments [18]. Of these, most investigators usually use the general measures that are applicable to different conditions and populations. Thus, the focus of this study is on developing a general instrument for measuring health literacy. In doing so we provided a short list of existing measures that includes the advantages and disadvantages of existing well-known general health literacy instruments (Table 1; for a more comprehensive list see [15, 18]).

**Table 1:** A short list of some selected instruments for measuring general health literacy

Name	Authors (publication Year), [ref]	Target	The most important advantages	The most important disadvantages
Rapid Estimate of Adult Literacy in Medicine (REALM)	Davis et l. (1991), [10].	Adults	Quick and easy to administer, short version available	Only measures reading ability, has problems when administering to patients with limited reading ability.
Test of Functional Health Literacy in Adults (TOFHLA)	Parker et al. (1995), [11].	Adults	Available in Spanish, German, French, and Italian. Short version available. Has been validated in several samples representing diverse populations.	The use of the instrument is limited to health service settings. The short-TOFHLA is only a test of reading comprehension and might prove useful as a screening instrument to identify patients with very limited reading ability rather than health literacy
Newest Vital Sign (NVS)	Weiss et al. (2005), [12].	Adults	Quick functional health literacy assessment that includes numeracy.	The scoring description lack precision. With high sensitivity, the NVS might misclassify patients with adequate health literacy, while the specificity might result in overestimating the percentage of patients with limited literacy
Screening Questions for Limited Health Literacy (SILS)	Morris et al. (2006), [19], Chew et al. (2008), [20]	People with limited literacy	Very easy and short (3 items)	Only measures reading, understanding and filling out medical forms
Medical Term Recognition Test (METER)	Rawson et al. (2010), [21].	Adults	Quick and easy to administer	It is a one-dimensional instrument
Health Literacy Skills Instrument (HLSI)	McCormack et al. (2010), [22].	Adults	Assesses multiple health literacy domains with a skills-based approach. Available in short form.	Primarily focusing on functional health literacy using different means such as documents, oral communication and Internet making it relatively difficult to admister
Health Literacy Assessment Using Talking Touchscreen Technology (Health LiTT)	Hahn, et al. (2011), [23].	Adults	Self-administered, computer adapted	Not able to distinguish higher levels of health literacy. Health literacy assessment might be influenced by computer literacy and skills
Canadian Self-Report Health Literacy Skills	Begoray and Kwan(2012), [24]	Adults	Short instruments that includes nine self-reported items	Uses very general items and cannot provide accurate estimation of health literacy
Health Literacy Questionnaire HLQ)	Osborne et al. (2013), [16].	Adults	Contains multiple domains of health literacy, relatively well developed	Not identified yet.
European Health Literacy Questionnaire (HLS-EU-Q47)	Sørensen et al. (2013), [17].	Age 15+	Comprehensive, available in more than 10 languages.	Developed in European context. However, ccurrently the Asian version also was developed.
HLS-EU-Q16	Sørensen et al. (2015), [25].	Age 15+	Comprehensive instrument	Developed in European context.
All Aspects of Health Literacy Scale (AAHLS):	Chinn and McCarthy, (2013) [26]	Age 15+	Measuring functional, communicative and critical health literacy	Although short, it is not useful for population studies since it might be confusing for people with limited education and literacy.

The aim of this study was to develop a rigorous and valid instrument for measuring health literacy for adults, yet easy to use, and multidimensional. Although the current study is not unique, perhaps could contribute to the existing knowledge on the topic as the instrument reported here was developed in a non-western country.

## Methods

### The conceptual framework

The core conceptual model presented by Ratzan and Parker inspired the current study’s conceptual framework of health literacy. The concept comprised the ability to obtain health information (access); understand health information (understanding); ability to assess and evaluate the health information (appraisal); and use the information to make a decision (apply or use health-related information) [27].

### Item generation

We used a similar methodology that previously was described in details elsewhere [28]. In brief, first a review of existing health literacy measures was conducted. The review was performed in 2012 and has since been updated. Consequently a panel of specialists in public health, health education/health promotion, health care management, mental health, oral health, maternal and child health, and community medicine was held. The selection of experts was based on their research interest, previous works on health literacy and managerial responsibility in the health care system. Panelists’ characteristics are presented in Table 2. Following ten sessions, of at least 3 h, an item pool of 400 statements was generated. Items were generated using brainstorming in each session and a member of the panel was responsible for listing the items. The panelists were limited to suggesting items related to the potential subscales (reading, access, understanding, appraisal, and behavioral intention). Then items were checked for duplicates and excluded when they were inconsistent with the intended subscales. Accordingly, the panel reduced the number of items to 66. Upon initial agreement on selected items a 5-point Likert scale (never =1, rarely =2, sometimes = 3, usually = 4, always = 5) was used to indicate the lowest to highest level of the respondents’ abilities. In the next step, content validity and face validity of the questionnaire was evaluated.

**Table 2:** The characteristics of panelists

Specialty	Age	Gender	Professional responsibility
Public Health	55	Male	Senior Researcher
Health Education	44	Male	Senior Researcher
Health Education	45	Female	Research Fellow
Health Education	43	Female	Research Fellow
Health Education/Health Promotion	50	Female	Senior Researcher
Health Education/Health Promotion	40	Female	Senior Researcher
Health Care Management	49	Female	Senior Researcher
Health Care Management	45	Female	Senior Researcher
Health Care Management	37	Female	Senior Researcher
Health Care Management	46	Female	Senior Researcher
Mental Health	50	Female	Senior Researcher
Oral Health	35	Male	Research Fellow
Community Medicine	45	Male	Senior Researcher
General Practitioner	45	Male	Research Fellow
Maternal and Child Health	51	Female	Senior Researcher

### Content validity

A group of 15 experts in public health was invited to assess the questionnaire. First, we asked the experts to check the items for relevance, clarity and simplicity on a 4-point Likert scale that comprised the options very relevant, relevant, relatively relevant, and not relevant. The experts were then asked to indicate necessity of each item by rating a 3-point Likert scale of essential, useful but not essential, and not essential. Next, experts were asked to comment on wording, and grammar. As a result, 19 items were removed and a provisional version of the questionnaire with 47 items was provided.

### Face validity

To determine the face validity of the questionnaire, 10 individuals aged 18–65 years were selected using a purposive sampling to verify the clarity, relevance and difficulty of each item. None of items were removed or changed at this stage and the Health Literacy Instrument for Adults (HELIA) was prepared for psychometric analysis.

### Main study

i. **Design and data collection**: Psychometric properties of the HELIA were examined by conducting a cross sectional study. In this regard, a random sample of adults aged 18 to 65 and living in Tehran was recruited. The sample size was calculated as the number of items in the questionnaire multiplied by 5 [29]. The participants completed the HELIA at their homes under trained interviewers’ supervision. Demographic data including the participants’ age, gender, education, job and source of health information, were also recorded.
ii. **Statistical analysis:** The structural validity and reliability of the HELIA were examined using Exploratory Factor Analysis (EFA) with varimax rotation and internal consistency respectively. The Kaiser-Meyer-Olkin (KMO) and Bartlett’s Test of Sphericity were used to determine the appropriateness of the sample for factor analysis [30, 31]. Eigenvalues above 1 and factor loadings greater than or equal to 0.50 were considered appropriate to verify the number of possible underlying factors. Furthermore, confirmatory factor analysis was performed while a five-factor model (access, reading, understanding, appraisal and behavioral intention) was specified. Several goodness-of-fit indicators including: chi-square ratio (χ2/df), goodness of fit index (GFI), the root mean square error of approximation (RMSEA), standardized root mean square residual (SRMR), normed fit index (NFI) and comparative fit index (CFI) were selected for reporting the analysis outcomes. The following thresholds were considered to verify the model’s goodness of fit: χ2/df < 2.0, CFI, NFI, NNFI, and GFI ≥ 0.90–0.95, SRMR ≤0.05–0.08, and RMSEA ≤0.05–0.06 [32–35]. Finally, the Cronbach’s alpha coefficient (acceptable level of 0.7) for each dimension and the whole scale was calculated to examine internal consistency [36]. Additionally the Intraclass Correlation Coefficient (ICC) was calculated by performing test-retest analysis to establish instrument stability. For this purpose, a convenience sample of 30 individuals aged 18 to 65 (mean age 34.9 ± 10.1, 18 female and 12 male) attending to a health center in Tehran was recruited. They have completed the questionnaire twice with a 1-week interval. The correlations of 0.75 or higher were considered satisfactory [37]. All statistical analyses were performed using SPSS version 17.0. The confirmatory factor analysis was performed using LISREL 8.8 software.

## Results

### Demographic characteristics

In all 336 adults aged 18 to 65 took part in the study and completed the questionnaire. Of these, 13 individuals were excluded due to incomplete response to the questionnaire. Thus, the data obtained from 323 participants were analyzed. The mean age of participants was 37.89 ± 13.31 years, 54% were females, and 39% were housewives. The characteristics of participants are shown in Table 3.

**Table 3:** Demographic characteristics of the study participants (*n* = 323)

	All	Male (***n*** = 147)	Female (***n*** = 176)
	No. (%)	No. (%)	No. (%)
**Age groups**			
18–30	117 (36.2)	54 (36.7)	63 (35.8)
31–42	90 (27.9)	38 (25.9)	52 (29.5)
43–54	67 (20.7)	30 (20.4)	37 (21.0)
55–65	49 (15.2)	25 (17.0)	24 (13.6)
**Education (years)**			
1–9	65 (20.1)	25 (17.0)	40 (22.7)
10–12	133 (41.2)	58 (39.5)	75 (42.6)
≥ 13	125 (38.7)	64 (43.5)	61 (34.7)
**Occupation**			
Employed	101 (31.3)	82 (55.8)	19 (10.8)
Student	45 (13.9)	25 (17.0)	20 (11.4)
Retired	29 (9.0)	21 (14.3)	8 (4.5)
Housewife	125 (38.7)	–	125 (71.0)
Unemployed	23 (7.1)	19 (12.9)	4 (2.3)
**Source of health information**^**a**^			
TV/Radio	173 (26.3)	74(25.3)	99(27.0)
Physicians/Health providers	161 (24.4)	76(26.0)	85(23.2)
Newspapers/Journals	93 (14.1)	44(15.1)	49(13.4)
Friends, Relatives	87 (13.2)	39(13.4)	48(13.1)
Internet	76 (11.5)	36(12.3)	40(10.9)
Book/Booklets/Pamphlets	45 (6.8)	12(4.1)	33(9.0)
Interactive voice response (IVR)	11 (1.7)	5(1.7)	6(1.6)
No answer	13 (2.0)	6(2.1)	7 (1.9)

^a^ Since the respondents could indicate several sources the numbers exceed the total sample size

### Factor structure

1. Exploratory factor analysis: The adequacy of sample size was confirmed by KMO and Bartlett’s Test of Sphericity (KMO = 0.919 and χ2 = 4101.78, *p* <  0.0001). The initial analysis indicated a 9-factor solution with eigenvalues greater than 1 that jointly accounted for 58.9% of the variance observed. After careful assessment, four factors were excluded for the following reasons:

a. There was a factor with two items more relevant to behavioral intension and thus the factor was excluded and the items conjugated to factor 1 (behavioral intension subscale).

b. Item loading on three factors did not satisfy the expected threshold. Examples of some low loading items read as follows: I can find health information about physical activity such as walking and exercise; I can understand health information on diet and obesity; I can fill-in medical forms when needed.

Thus after deletion of the low loading items (with one exception), eventually 33 items were loaded on 5 factors: access to information (4 items), reading (6 items), understanding (7 items), appraisal (4 items) and behavioral intention (12 items), that jointly accounted for 52.9% of the variance observed. The detailed results are shown in Table 4 [Additional file 1].

**Table 4:** The results obtained from exploratory factor analysis for the HELIA

Item	F1	F 2	F 3	F 4	F 5
Reading educational materials about health (booklets, pamphlets, leaflets) is easy for me.	0.172	0.061	0.201	0.163	**0.656**
Reading written instructions from doctors, dentists and health workers about my illness is easy for me.	0.148	0.154	0.287	0.086	**0.551**
Reading medical and dental forms (such as admissions, consent, filing, etc. in hospitals and medical centers) is easy for me.	0.069	0.307	0.217	0.010	**0.700**
Reading leaflets and instructions for laboratory testing, ultrasound or radiology is easy for me.	0.168	0.186	0.077	0.117	**0.701**
I can find health information from different sources when I need such information.	0.035	0.121	**0.641**	0.125	0.244
I can find health information about healthy eating.	0.125	0.137	**0.683**	0.218	0.159
I can find health information on mental health such as depression and stress.	0.090	0.072	**0.754**	0.112	0.044
I can find health information about a specific disease when I need to.	0.156	0.258	**0.691**	(0.058)	0.053
I can find health information for some health problems and diseases such as high blood pressure, high blood sugar and high lipid levels.	0.204	0.197	**0.557**	0.079	0.241
I can find health information about harmful effects of tobacco and smoking.	0.191	0.060	**0.445**	0.254	0.204
I can understand the recommendations for a healthy diet.	0.295	**0.587**	0.230	0.207	0.097
I can understand when my physician explains about my illness.	0.172	**0.726**	0.129	0.182	0.048
I can understand the meaning when reading medical forms (such as admissions, consents, filings, etc.) in hospitals and health centers.	0.152	**0.616**	0.170	0.147	0.314
I can understand signage guidelines in hospitals, clinics and health centers.	0.201	**0.574**	0.069	0.227	0.308
I can understand drug information on labels.	0.239	**0.698**	0.114	0.163	0.122
I can understand the risks, and benefits of drugs prescribed by my physician.	0.204	**0.743**	0.183	0.110	0.035
I can understand written information before testing, ultrasound or radiology.	0.150	**0.627**	0.158	0.110	0.255
I can evaluate health-related information on the Internet.	0.127	0.136	0.185	**0.603**	0.105
I can evaluate health-related information broadcast on television and radio.	0.251	0.192	0.114	**0.764**	0.104
I can assess the accuracy of health-related recommendations I receive from relatives and friends.	0.238	0.276	0.198	**0.680**	0.057
I can communicate trusted health information to others.	0.253	0.303	0.079	**0.587**	0.175
When facing an illness, I know where to go or with whometo speak.	**0.510**	0.230	0.333	0.044	0.036
When physician suggests that I should take antibiotic capsules three times a day I know that I should take one tablet every 8 h.	**0.579**	0.244	0.108	0.216	0.099
I do not cut my medications without my physician’s permission, even if symptoms disappear.	**0.666**	0.117	0.176	(0.005)	0.064
If anyone from my first-degree relatives develops cancer (such as prostate, breast, cervix, colon, etc.), I see a doctor to examine me.	**0.665**	0.020	(0.018)	0.212	0.129
I avoid doing or eating things that increase my blood pressure.	**0.644**	0.213	0.097	0.190	0.069
I visit my physician for regular checkups.	**0.698**	(0.063)	0.145	0.116	0.139
I am health-conscious in any situation.	**0.651**	0.121	0.068	0.139	0.056
If needed, I ask my physician or health care team questions about my disease.	**0.590**	0.338	0.118	0.142	0.075
I buy dairy products (milk, yoghurt, cheese, etc.) according to their fat percentage.	**0.637**	0.235	0.155	(0.065)	0.085
I avoid using substances that increase my weight.	**0.620**	0.157	0.058	0.043	0.193
I use a seat belt when driving.	**0.608**	0.289	0.051	0.318	0.101
I consider the food labels when shopping.	**0.649**	0.180	0.084	0.276	0.046
**Eigenvalue**	**5.450**	**4.040**	**3.113**	**2.517**	**2.368**
**Explained Variance (%)**	**16.514**	**12.243**	**9.434**	**7.627**	**7.176**
**Cumulative Variance (%)**	**16.514**	**28.757**	**38.192**	**45.819**	**52.995**

Factor 1. Decision-making/ behavioral intention, Factor 2. Understanding, Factor 3. Access to information, Factor 4. Appraisal, Factor 5. Reading

**Fig. 1:**
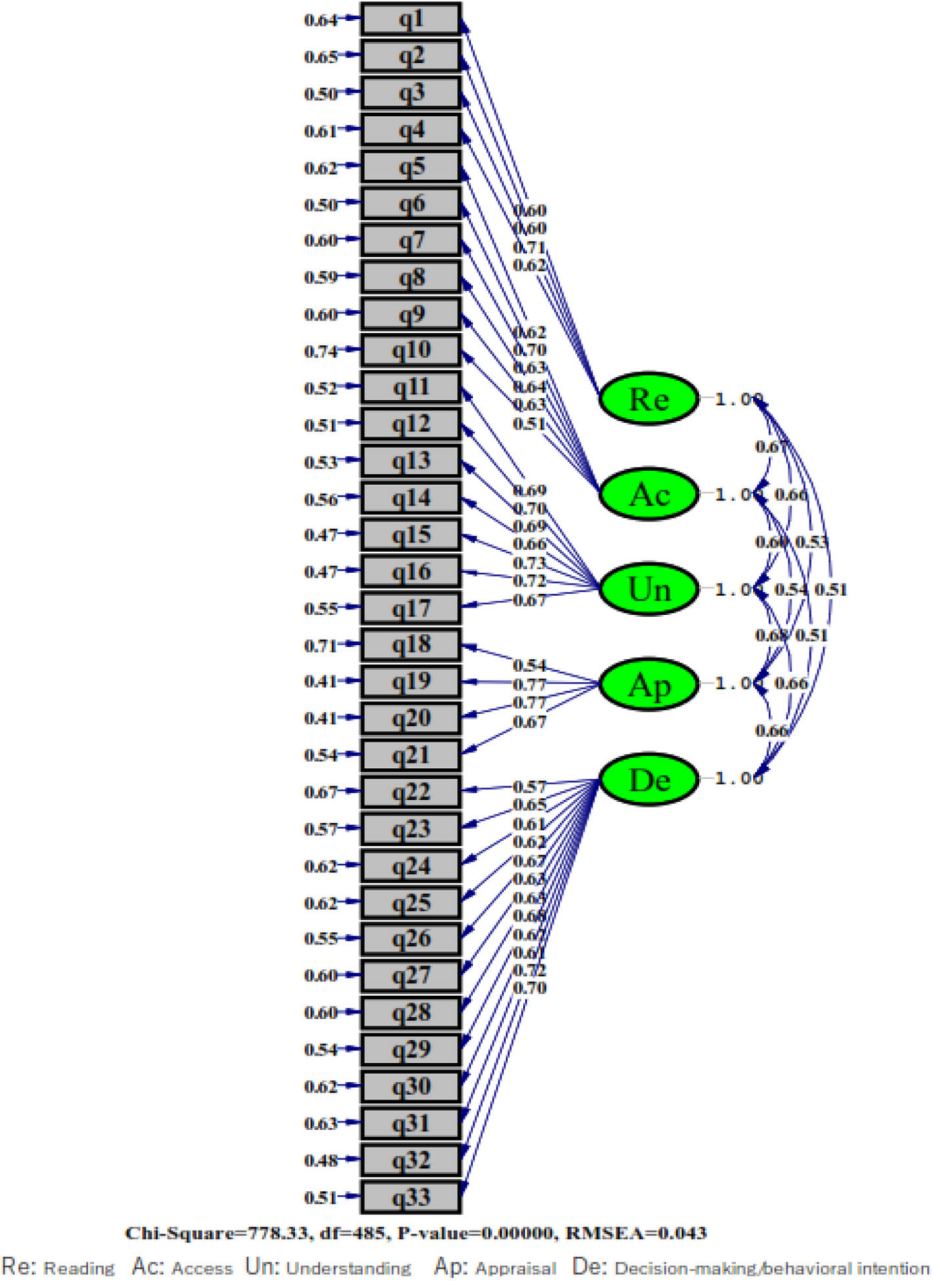
The result obtained from confirmatory factor analysis for the HLIA

2. Confirmatory factor analysis: The result obtained from the confirmatory factor analysis is depicted in Fig. 1. The results provided a good fit to the data. The fit indexes were as follows: χ^2^ = 778.33; χ^2^/df = 1.60; SRMR = 0.049; RMSEA (90% CI) = 0.043 (0.038–0.049); CFI = 0.98; NFI = 0.95; and NNFI = 0.98; GFI = 0.87 (Table 5). The correlations between latent factors are also presented in Table 6.

**Table 5:** Fit indices and their acceptable thresholds in confirmatory factor analysis

Fit Index	Values
**Chi-Square χ2**	815.90 (*P* = 0.001)
**Relative χ2** (χ2/df)	1.68
**RMSEA** (Root Mean Square Error of Approximation)	0.046
**GFI** (Goodness-of-fit index)	0.87
**SRMR** (Standardized root mean square residual)	0.053
**NFI** (Normed fit index)	0.95
**NNFI** (Non-normed fit index)	0.98
**CFI** (Comparative fit index)	0.98

**Table 6:** Correlations between latent factors obtained form confirmatory factor analysis

	Re	Ac	Un	Ap	De
**1.** Reading (Re)	1				
**2.** Access to information (Ac)	0.67	1			
**3.** Understanding (Un)	0.66	0.60	1		
**4. A**ppraisal (Ap)	0.53	0.54	0.68	1	
**5.** Decision making/behavioral intention (De)	0.51	0.51	0.66	0.66	1

### Reliability

Reliability was assessed by estimating the Cronbach’s alpha coefficient. The results showed that all factors had acceptable internal consistency. The Cronbach’s alpha coefficient for each subscale and the questionnaire as a whole ranged from 0.72 to 0.89 (Table 7). Further analysis indicated that the alpha coefficient could not be improved if any further items deleted. The stability of the HELIA and its sub-scales as measured by the Intraclass Correlation Coefficient (ICC) was also found to be satisfactory. All ICCs were above acceptable threshold (Table 7).

**Table 7:** Cronbach’s α coefficient and ICC for the HELIA and its subscales

Domain	Number of items	Cronbach’s α coefficient	Intraclass Correlation Coefficient (ICC)
**Reading**	4	0.72	0.86
**Access to information**	6	0.79	0.91
**Understanding**	7	0.86	0.81
**Appraisal**	4	0.77	0.76
**Decision making/behavioral intention**	12	0.89	0.87
**The scale**	33	0.93	0.84

### Health literacy

The mean health literacy score for the study sample was 76.3 (SD = 15.1). Overall 78.6% of the respondents showed adequate health literacy while the remaing 21.4% had limited health literacy. There was no significant difference in health literacy among male and female respondents (*P* = 0.33), although women scored higher compared to male respondents (77.5 vs. 74.9 respectively). However, health literacy score was sginficatly different among people who differed in age, education, and employment status as expected. These are presented in Table 8. The scoring manual for the HELIA is supplemented (Additional file 2).

**Table 8:** Health literacy based on demographic variables

	Health lieracy score	Limited health literacy	Adequate health literacy	
	Mean (SD)	No. (%)	No. (%)	P^**a**^
**Age**				0.045
18–30	75.8 (13.7)	23 (33.3)	94 (37.0)	
31–42	80.4 (12.6)	12 (17.4)	78 (30.7)	
43–54	74.1 (15.0)	20 (29.0)	47 (18.5)	
55–65	73.0 (20.6)	14 (20.3)	35 (13.8)	
**Gender**				0.33
Female	77.5 (14.8)	34 (49.3)	142 (55.9)	
Male	74.9 (15.3)	35 (50.7)	112 (44.1)	
**Education (years)**				< 0.001
1–9	66.8 (18.4)	29 (42.0)	36 (14.2)	
10–12	76.5 (12.2)	24 (34.8)	109 (42.9)	
13 ≥	81.1 (13.7)	16 (23.2)	109 (42.9)	
**Employment**				0.004
Employed	78.6 (12.3)	16 (23.2)	85 (33.5)	
Student	79.3 (12.4)	5 (7.2)	40 (15.7)	
Retired	77.2 (17.7)	6 (8.7)	23 (9.1)	
Housewife	75.0 (15.9)	31 (44.9)	94 (37.0)	
Unemployed	66.6 (19.4)	11 (15.9)	12(4.7)	
**Total**	76.3 (15.1)	69 (21.4)	254 (78.6)	

^a^ Dervided from Chi-square

## Discussion

The findings showed that the HELIA is a valid instrument for measuring health literacy among adult populations and could be considered as a useful measure along with other recently developed instruments [16, 17, 38, 39]. However, it is important to note that although the methods we used were scientific, they were traditional and not as strong as the methods of two recently developed measures [16, 17]. For instance, for development of the Health Literacy Questionnaire (HLQ) Osborne et al. followed a validity-driven method that involved systematic grounded approaches in which existing theories were not considered until later in the development process of the questionnaire [16]. In fact, they first focused on individuals’ and professionals’ lived experiences and then used the definition of health literacy proposed by the World Health Organization.

The HELIA has a multidimensional structure that can be easily used for public health purposes. Although not identical to the HELIA, the European Health Literacy Survey is also a relatively comprehensive instrument. It has two sections, a core health literacy section and a section on determinants and outcomes associated with health literacy. Indeed in the European Health Literacy Survey model ‘health literacy refers to an evolving set of competencies that do not remain static over time and can be regarded as a means to an end rather than a fixed state, to which a person should aspire’ [17]. However, the very well known and popular instruments cover only a few dimensions of health literacy and none assess the broad range of abilities such as access to information, reading, understanding, appraisal, and decision making (behavioral intention). For example, the Test of Functional Health Literacy in Adults (TOFHLA) assesses only reading, comprehension and numeracy skills and it seems that completion of this test would be difficult for those who are not well educated to complete. The Rapid Estimate of Adult Literacy in Medicine (REALM), another well-known scale for measuring health literacy, is also examine only reading and recognition of medical words. Although it is a brief and easy to use instrument, it comprises just one dimension.

The HELIA contains five subscales (dimensions), which we believe is an important feature of this instrument covering the basic concept and constructs that build the meaning of health literacy. Additionally the items that included in the HELIA are relevant to public health in general and to healthy life styles in particular. In fact, underlying concepts included in the instrument cover the three most important global public health topics, which are issues related to cardiovascular diseases (nutrition items), cancers and accidents. These topics were arranged in a way that people with both limited literacy and a high level of education could easily respond to items. We did not want to test people’s knowledge but rather were interested in examining skills relevant to health literacy. Indeed, we believe a range of people with education ranging from primary to higher could relate the items to themselves and provide honest responses to the questionnaire. However, one should notice that the current version of the HELIA has some limitations. For instance, because numeracy skill is an important issue in a health care context, it is necessary to add a few more items to the questionnaire.

Nevertheless all these efforts should be greeted because relying on one measure might not properly help policy and practice. It is argued that since social, environmental and cultural factors influence health literacy in different populations, the need for integration of definitions and models of health literacy are essential [40]. In this respect, it seems that items addressing numerical literacy and media literacy might also be necessary in new versions of existing health literacy instruments including the HELIA. Furthermore, to measure how valid the invented instrument is, it is necessary to compare the results of measurements with other recognized instruments and to show that at least some scales show comparable results (concurrent or criterion validity). The current study did not include such analysis and future studies should therefore employ a previously validated instrument and report on concurrent or criterion validity. One more limitation is the fact that the instrument was tested in one location using a cross-sectional approach and stability (test-retest analysis) was examined in a separate sample. Finally, it is important to remember that no external assessment was applied for the HELIA to objectively assess different skills. For instance when the respondent says that she or he is always understands the content, all the answers the respondents give are self-estimated skills and not objective ones. Thus, some measures should be integrated into the questionnaire to assess actual skills.

## Conclusion

The results showed that the Health Literacy Instrument for Adults (HELIA) is a valid and reliable measure for assessing health literacy among adults.

## Supplementary information

**Additional file 1.** The HELIA English version.

**Additional file 2.** The scoring manual for the HELIA.

## Data Availability

The data is available from the corresponding author upon reasonable request. The Persian version of the HELIA also will be available from the corresponding author.

## Abbreviations

ICC: Intraclass correlation coefficient
HELIA: Health Literacy Instrument for Adults
REALM: Rapid Estimate of Adult Literacy in Medicine
TOFHLA: Test of Functional Health Literacy in Adults
NVS: Newest Vital Sign
HLQ: Health Literacy Questionnaire
HLS-EU-Q47: 47-item European Health Literacy Survey Questionnaire
HLS-EU-Q16: 16-item European Health Literacy Survey Questionnaire
AAHLS: All Aspects of Health Literacy Scale
