# Supplementary material for: Development and validation of a short and easy-to-use instrument for measuring health literacy: the Health Literacy Instrument for Adults (HELIA)
Source: BMC Public Health. 2020 May 12;20:656. doi: 10.1186/s12889-020-08787-2 (PMC7216550; doi:10.1186/s12889-020-08787-2)
Supplement: Supplementary file 1 — Additional file 1. The HELIA English version. [file 12889_2020_8787_MOESM1_ESM.pdf]

Health Literacy Instrument for Adults (HELIA)

Dear respondent,

This questionnaire deals with your perceived abilities and competencies on obtaining and using health information. For each question, put a check mark or cross in the box in front of the answer that best describes your abilities or skills. Please answer all questions.

**Reading**	**Never**	**Rarely**	**Sometimes**	**Usually**	**Always**
1. Reading educational materials about health (booklets, pamphlets, leaflets) is easy for me.	1	2	3	4	5
2. Reading written instructions from doctors, dentists and health workers about my illness is easy for me.	1	2	3	4	5
3. Reading medical and dental forms (such as admissions, consent, filing, etc. in hospitals and medical centers) is easy for me.	1	2	3	4	5
4. Reading leaflets and instructions for laboratory testing, ultrasound or radiology is easy for me.	1	2	3	4	5
**Access to information**	**Never**	**Rarely**	**Sometimes**	**Usually**	**Always**
5. I can find health information from different sources when I need such information.	1	2	3	4	5
6. I can find health information about healthy eating.	1	2	3	4	5
7. I can find health information on mental health such as depression and stress.	1	2	3	4	5
8. I can find health information about a specific disease when I need to.	1	2	3	4	5
9. I can find health information for some health problems and diseases such as high blood pressure, high blood sugar and high lipid levels.	1	2	3	4	5
10. I can find health information about harmful effects of tobacco and smoking.	1	2	3	4	5
**Understanding**	**Never**	**Rarely**	**Sometimes**	**Usually**	**Always**
11. I can understand the recommendations for a healthy diet.	1	2	3	4	5
12. I can understand when my physician explains about my illness.	1	2	3	4	5
13. I can understand the meaning when reading medical forms (such as admissions, consents, filings, etc.) in hospitals and health centers.	1	2	3	4	5
14. I can understand signage guidelines in hospitals, clinics and health centers.	1	2	3	4	5
15. I can understand drug information on labels.	1	2	3	4	5

16. I can understand the risks and benefits of drugs prescribed by my physician.	1	2	3	4	5
17. I can understand written information before testing, ultrasound or radiology.	1	2	3	4	5
**Appraisal**	**Never**	**Rarely**	**Sometimes**	**Usually**	**Always**
18. I can evaluate health-related information on the Internet.	1	2	3	4	5
19. I can evaluate health-related information broadcast on television and radio.	1	2	3	4	5
20. I can assess the accuracy of health-related recommendations I receive from relatives and friends.	1	2	3	4	5
21. I can communicate trusted health information to others.	1	2	3	4	5
**Decision-making/ behavioural intention**	**Never**	**Rarely**	**Sometimes**	**Usually**	**Always**
22. When facing an illness, I know where to go or with whom to speak.	1	2	3	4	5
23. When physician suggests that I should take antibiotic capsules three times a day, I know that I should take one tablet every 8 hours.	1	2	3	4	5
24. I do not cut my medications without my physician’s permission, even if symptoms disappear.	1	2	3	4	5
25. If anyone from my first-degree relatives develops cancer (such as prostate, breast, cervix, colon, etc.), I see a doctor to examine me.	1	2	3	4	5
26. I avoid doing or eating things that increase my blood pressure.	1	2	3	4	5
27. I visit my physician for regular checkups.	1	2	3	4	5
28. I am health-conscious in any situation.	1	2	3	4	5
29. If needed, I ask my physician or health care team questions about my disease.	1	2	3	4	5
30. I buy dairy products (milk, yoghurt, cheese, etc.) according to their fat percentage.	1	2	3	4	5
31. I avoid using substances that increase my weight.	1	2	3	4	5
32. I use a seat belt when driving.	1	2	3	4	5
33. I consider the food labels when shopping.	1	2	3	4	5

Thank you for completing the questionnaire

© Montazeri et.al, 2017

Health Metrics Research Center, Iranian Institute for Health Sciences Research, ACECR, Tehran, Iran.
