# Supplementary material for: Development and validation of a short and easy-to-use instrument for measuring health literacy: the Health Literacy Instrument for Adults (HELIA)
Source: BMC Public Health. 2020 May 12;20:656. doi: 10.1186/s12889-020-08787-2 (PMC7216550; doi:10.1186/s12889-020-08787-2)
Supplement: Supplementary file 2 — Additional file 2. The scoring manual for the HELIA. [file 12889_2020_8787_MOESM2_ESM.pdf]

Manual for scoring the HELIA

	Number of items	Minimum possible raw score	Maximum possible raw score
**Reading**	**4 (item 1-4)**	**4**	**20**
**Access**	**6 (item 5-10)**	**6**	**30**
**Understanding**	**7 (item 11-17)**	**7**	**35**
**Appraisal**	**4 (item 18-21)**	**4**	**20**
**Decision-making/ behavioral intention**	**12 (item 22-33)**	**12**	**60**
**Total**	**33 (item 1-33)**	**33**	**165**

To calculate each subscale or total score for the HELIA, first add item scores to achieve raw scores and then linearly transfer it to a score from 0 to 100 using the following formula.

$$\text{Score}=\frac{\text{Raw score}-\text{Minimum possible raw score}}{\text{Maximum possible raw score}-\text{Minimum possible raw score}}*100$$

Health literacy category levels*

The HELIA scores		Categories	
**Minimum**	**Maximum**	**Rank**	**Levels**
0	50	Inadequate	Limited
50.1	66	Somewhat inadequate	
66.1	84	Sufficient	Adequate
84.1	100	Excellent	

*__Adapted from:__ Sørensen K, Pelikan JM, Röthlin F, Ganahl K, Slonska Z, Doyle G, Fullam J, Kondilis B, Agrafiotis D, Uiters E, Falcon M, Mensing M, Tchamov K, van den Broucke S, Brand H; HLS-EU Consortium. Health literacy in Europe: comparative results of the European health literacy survey (HLS-EU). European Journal of Public Health 2015; 25(6): 1053–58.

© Montazeri et.al, 2017.

Health Metrics Research Center, Iranian Institute for Health Sciences Research, ACECR, Tehran, Iran.
